# Becoming Healthier without Paying More? Experimental Evidence from the Impact of Multiple Traffic Lights on Chinese College Students

**DOI:** 10.3390/nu16132124

**Published:** 2024-07-03

**Authors:** Jing Lin, Tingyu Wang, Wen Lin

**Affiliations:** 1 Jinshan College of Fujian Agriculture and Forestry University, Fuzhou 350001, China; ssagef@sina.cn; 2 China Academy for Rural Development, School of Public Affairs, Zhejiang University, Hangzhou 310058, China; 22322413@zju.edu.cn

**Keywords:** traffic light label, nutrition, food choices, cost

## Abstract

The prevalence of overweight and obesity among Chinese residents has become a pressing public health concern. The UK Multiple Traffic Light labeling system, known for its user-friendly design, has demonstrated success in promoting healthier food choices. This paper presents novel findings from a randomized controlled experiment assessing the impact of traffic light labeling on Chinese consumers’ food choices. Results indicate that the label significantly reduces the intake of calories, fat, carbohydrates, and sodium without increasing the economic costs of food choices. This study contributes empirical evidence to the effectiveness of traffic light labeling in China, with implications for the country’s approach to front-of-pack nutrition labeling.

## 1. Introduction

The global prevalence of obesity is on the rise due to rapid socio-economic development and significant lifestyle changes among consumers. For example, in 2020, the rates of overweight and obesity among Chinese residents aged 18 years and above were 34.3% and 16.4%, respectively, as reported by the Chinese Nutrition Society in 2021. This phenomenon poses a critical risk factor for cardiovascular disease, diabetes, hypertension, and cancer, highlighting the need for health public interventions.

Nutrition labeling is a crucial tool for enhancing individuals’ healthy food choices via the provision of nutritional information on food products [1,2]. Specifically, front-of-pack nutrition labeling communicates the nutritional quality of food products using simple icons, enhancing consumers’ understanding of food nutritional information and serving as a convenient supplement to traditional back-of-pack nutritional labels [3,4,5]. One of the most popular front-of-pack labels refers to the traffic light nutrition labeling scheme. The traffic light nutrition-labeling scheme, pioneered by the UK Food Standards Agency, utilizes a color-coded system to denote the levels of various nutrients. Each nutrient is represented by a color—green for low, amber for medium, and red for high levels. The multiple traffic light (MTL) label has been adopted by many developed countries, including the United States, the United Kingdom, France, Switzerland, Canada, and Singapore [6,7,8,9], and some developing countries like Peru, Argentina, and Bulgaria [10]. No other studies have tested the effectiveness of the MTL scheme in improving individuals’ healthy food choices in China despite a large portion of the population being overweight or obese.

In China, the only mandatory nutrition label refers to nutritional content tables on the back of the package [2]. Studies have indicated that the effectiveness of the back-of-pack nutritional fact panel is limited in encouraging consumers to make healthy choices due to the challenge residents face in understanding the label [11,12,13]. On the other hand, China, being one of the world’s largest food markets, has seen a significant rise in the consumption of prepackaged foods due to their convenience and easy storage [14]. Studies reveal that processed foods contribute substantially to dietary elements that need to be restricted [15]. The excessive consumption of prepackaged foods high in sugar, salt, and fat is closely linked to nutrition-related chronic diseases such as obesity and hypertension [16,17,18,19]. Hence, there is an urgent need to design and implement more straightforward and user-friendly labels to guide Chinese residents to consume healthier food.

This study investigates the impacts of the MTL scheme on the healthiness and expenditure of individual food choices using a laboratory experiment in China. The impacts of the MTL scheme on food consumption are not conclusive in the literature. Relevant research has demonstrated the effectiveness of MTL in improving the healthfulness of consumers’ food choices compared to other front-of-pack nutrition labeling schemes [20,21,22,23]. Research by Crosetto et al. [24] suggests that nutritional improvements resulting from labeling may come at an economic cost. However, this cost does not exhibit regressive effects compared to other policies such as taxes and subsidies, as indicated by Muller et al. [24]. However, another strand of the studies has found that the MTL scheme turns out to be useless in affecting consumer food choices [25,26]. While many consumers appreciated the addition of signal light colors to the MTL, some experienced confusion about the label [27]. Moreover, the MTL performs weaker than warning labels in encouraging individual purchases of healthier foods [28].

This study examines the impact of the multiple traffic light nutrition labels on individual choices of prepackaged food products, with a laboratory randomized control experiment on Chinese consumers. In particular, we focus on college students who are one of the major consumers of prepacked food products. Studies have found that the behavior of students closely mirrors that of non-students in experimental settings. We aim to add to the literature on the effectiveness of the MTL scheme by offering additional empirical evidence on China, the largest food market worldwide.

## 2. Survey Design

This section describes the design of the randomized experiment, traffic light nutritional labels, and the questionnaire in this study.

### 2.1. Experimental Design

To investigate the impacts of traffic light nutritional labels on food choices, we used a between-subject design and randomly assigned subjects into one of two groups: a control group and a labeled group. A between-subject experiment offers several advantages. The controlled setting, careful selection of food products, and randomization of subjects control for confounding factors that could otherwise impact the effectiveness of traffic light labels.

Both the control and labeled groups completed their food purchases within a simulated food shopping platform. The only difference between the two groups lies in the fact that all food products in the labeled group were accompanied by corresponding traffic light labels, adhering to the standards outlined in Table 1. Relevant studies have demonstrated that simulated food purchasing environments serve as valuable and practical tools for studying the effects of health interventions, leading to results that closely parallel real-world findings [29,30,31,32].

**Figure 1:**
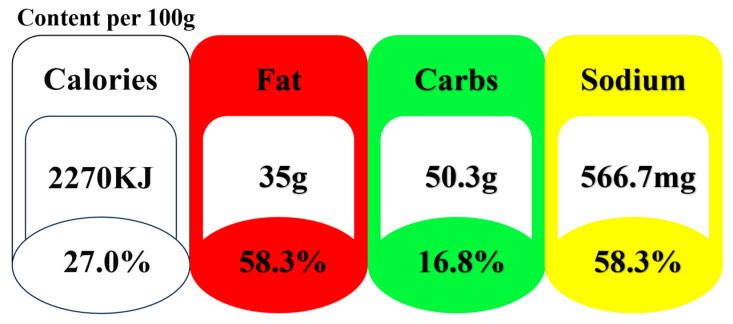
**A sample of traffic light nutrition label.** Notes: This white panel indicates the amount of energy per 100 g in a food item. The red, green, and yellow panels refer to the amount of fat, carbohydrates, and sodium.

**Figure 2:**
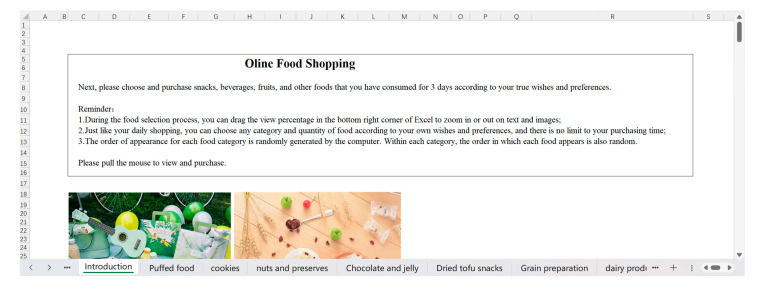

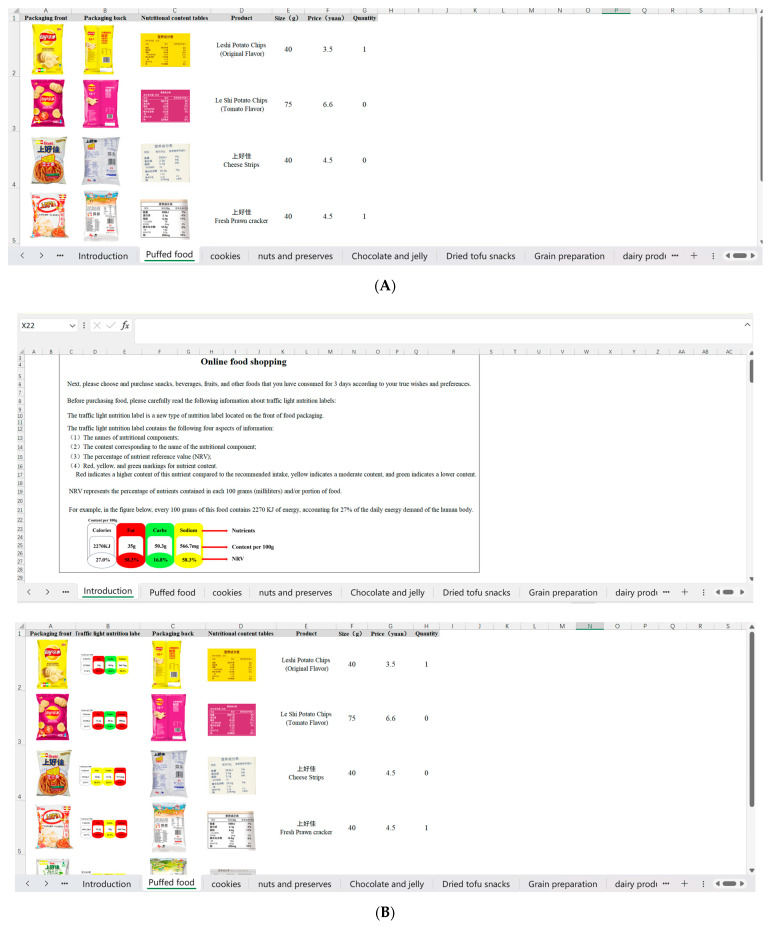
(**A**). **The online shopping platform of the control group (Traffic = 0)**. (**B**). **The online shopping platform of the treatment group** (Traffic = 1). Notes: the only difference in online shopping platforms between the control group and the treatment group is that the latter included the explanation on traffic nutrition labels, and each food product has been attached with a corresponding traffic nutrition label.

The simulated food shopping platform of this study included a total of 12 categories and 85 food products, identical for the control and labeled groups. The 12 food categories include chips, bread and pastries, cookies, nuts, jellies, chocolates and marinated seaweeds, dried beans, cereal mixes, dairy products, drinking water and soda, carbonated beverages, fruit juices and tea beverages, and fruits. The food categories and products in the platform were selected from the best-selling prepackaged food items within each respective product category, both online and offline, in the experimental city’s grocery stores. In addition to evaluating sales volume, the selection of food products took into account various attributes, such as nutritional content, price, and taste. This approach is tailored to closely replicate the scenarios faced by consumers in their real food shopping experiences. Figure 1 presents the food shopping platform of the control group, where the front and back packaging, price, name, and size of each product were shown to consumers. The food shopping platform of the labeled group was built on the control group’s platform, with the addition of a traffic light nutrition label for each product (Figure 2). In both platforms, consumers made their choices by selecting the quantity of food products through a click-based interaction.

### 2.2. Traffic Light Label Design

In the absence of official traffic light labels in China, this study crafted the labels by drawing upon official documents and the related literature. These sources include the General Principles for Nutrition Labeling of Prepackaged Foods in China, the Guidelines for Nutrition Claims and Nutrient Functional Claims of Foods in China [33], the United Kingdom’s Traffic Nutrition Labels from the UK Food Standard Agency, and the study of Seidelmann et al. [34].

The MTL scheme, initially devised by the UK Food Standards Agency, employs a color-coded system for various nutrients. In this scheme, the color signifies low (green), medium (amber), or high (red) levels. The green threshold aligns with the standards outlined in Regulation No. 1924/2006 for making low-level nutrition claims. Nutrients receive a red label if their content surpasses 25% and 12.5% of the maximum recommended daily intake for adults in solid and liquid foods, respectively. The color coding is established on a per 100 g basis for solid foods and per 100 mL for liquid foods.

Table 1 outlines the precise standards governing our MTL scheme, which is adapted based on the UK’s version. These labels provide information on four nutritional elements—calories, fat, carbohydrates, and sodium—for a range of items, including solid foods like cookies, chips, and chocolate, and liquid foods such as fruit juice, yogurt, and soda. In particular, if the fat content per 100 g of solid food is 3 g or below, or the fat content per 100 milliliters of liquid food is 1.5 g or less, the product is classified as low fat, and the fat panel is marked with the color green. Conversely, if the fat content per unit of a product exceeds 25% of the reference level recommended by the Dietary Nutrient Reference Intake for Chinese Residents, the product is designated as high fat, and its traffic light label includes a red fat panel. Regarding sodium, we define a food item as low in sodium when the sodium content per 100 g of solid food or 100 mL of liquid food is 120 milligrams or less. Conversely, a food product is classified as high sodium and marked with a red fat panel if the sodium content equals or exceeds 600 milligrams per unit of solid food or 300 milligrams per unit of liquid food. While the UK version incorporates sugar content into MTL, China does not mandate the disclosure of sugar content on food products. Instead, China mandates the disclosure of carbohydrates in nutrition labeling. Thus, this study includes carbohydrates instead of sugars in the MTL scheme. Following Seidelmann et al. [34], foods with less than 40% of calories per 100 g or 100 milliliters derived from carbohydrates are categorized as low carbohydrate (green). Conversely, foods with more than 70% carbohydrates are designated as high carbohydrate (red), while those with carbohydrate content between 40% and 70% are labeled as medium carbohydrate (yellow). For an illustration of the traffic light label, please refer to Figure 1.

### 2.3. Questionnaire Design

To better understand consumers’ food attitudes and choice behavior, we design survey questionnaires before food purchases in the simulated platform. The questionnaire included information on sociodemographic characteristics, usual food purchase and consumption, nutritional knowledge level, and psychological status. First, the sociodemographic part asked for subjects’ gender, age, residence, grade, height, weight, household size, pre-tax annual household income from all sources, need to lose or gain weight, etc. The food purchase and consumption part included monthly food expenditures, frequency of eating out and ordering food delivery, food allergies, and chronic diseases. The third part, nutritional knowledge level, was elicited by the General Nutritional Knowledge Questionnaire (GNKQ). GNKQ was first developed by Parmenter and Wardle in the UK in 1999 and then adopted in other countries, such as Australia [35], Turkey [36], and Japan [37]. This scale performs well in distinguishing participants’ nutrition knowledge levels and predicting their food choice behavior [35,38,39,40,41,42]. Last, cognitive depletion would have a strong effect on food choices [43]. So, we assess the subjects’ stress and anxiety by using The Depression–Anxiety–Stress Scale (DASS-21). DASS-21 is a streamlined version of the DASS developed by Lovibond et al. [44]. While keeping the dimensions of the original scale unchanged, seven items were retained for each of the three subscales of depression, anxiety, and stress in order to improve the efficiency of recognizing and assessing the symptoms of the corresponding mood disorders [45]. Studies have shown that DASS-21 is more suitable than the full version of the DASS [46,47,48,49]. To ensure the quality of responses, we include two attention checks in the questionnaire. Table 2 presents the description of the survey questions.

## 3. Survey Procedure

There are three steps in our entire survey.

Upon arrival, participants received a hard copy of the survey instructions and were instructed to thoroughly read them. We further elucidated the instructions and demonstrated to participants how to make purchases on the platform. Participants were encouraged to ask any questions regarding the instructions or the survey. After ensuring clarity on the entire experimental process and details, participants proceeded to sign a consent form, signifying their understanding of the experiment’s content, benefits, and potential risks. Specifically, participants were told that they would receive a payment of 15 RMB after finishing the food purchase and questionnaire.

Next, participants proceed with the online questionnaire.

After completing the questionnaire, participants entered the food shopping platform to make food choices. In this segment, participants were tasked with choosing any food products in the platform for their 3-day consumption. There was no time constraint for the subjects, and they could choose any number of food products they desired. To ensure incentive compatibility in food choices, participants were notified that *one product from their shopping list would be randomly chosen for an actual transaction*, and they would be required to pay the corresponding price. The selected product would then be delivered to them within 24 h. Therefore, food choices made by respondents were subject to individual preferences and budget constraints, close to their actual food purchases.

## 4. Data and Description

We recruited participants from a Chinese university in April and May 2021. Before implementing the survey and experiment, we obtained ethical approval from the university. We released the research recruitment information through the on-campus forum popular among college students, and individuals joined the study voluntarily by completing an enrollment questionnaire, which assessed their eligibility based on criteria such as (1) being over 18 years old, and (2) having purchased food products last month. Finally, a total of 100 qualified college students were randomly assigned to one of the two groups, with 47 in the control group and 53 in the labeled group. (The size of our control and labeled groups is consistent with other studies using between-subject designs [50,51]. The average survey duration was 20 min. Table 2 details the variables and their corresponding data.

## 5. Empirical Method and Results

### 5.1. Empirical Model

We used ordinary least-square models to examine the effects of traffic light labels on consumer food choices. The model specification is:

(1) $$Y_{i}=\alpha_{0}+\alpha_{1}Traffic_{i}+\alpha_{2}X_{i}+\varepsilon_{i}$$

where i denotes individual i, $Y_{i}$ refers to the dependent variables, including the natural logarithm of the total calories, fat, carbohydrates, sodium, and expenditure of the food basket selected by individual i. The key independent variable, $Traffic_{i}$, is a dummy variable that equals one if individual i was in the labeled group, and zero otherwise. $X_{i}$ is a vector of control variables, accounting for the impacts of socio-demographics, usual food purchase and consumption habits, and health and nutrition status. The definitions and descriptions of variables are outlined in Table 2 and Table 3.

### 5.2. Baseline Effects

Table 3 presents a comparison of differences between the control and labeled groups. In terms of socio-demographic characteristics, approximately 64% of the participants in this experiment were female, with an average age of 21 years and the majority residing in urban or suburban areas. The subjects have an average height of 1.66 m and an average weight of 57.65 kg. Approximately 8% self-reported as overweight, with over half expressing a desire to lose weight. Around 11% of participants reported being lactose intolerant or having allergies, and about 6.67% suffered from chronic conditions such as gastroenteritis and hypoglycemia. Most subjects belonged to families consisting of three-to-four people, with their families reporting an annual income falling within the range of RMB 50,000–120,000. Regarding daily dietary consumption, participants had an average monthly dietary expenditure ranging from RMB 1000 to 1500. The average weekly frequency of ordering takeaways or eating out for the participants was approximately 26%. Moreover, while most participants demonstrated a certain level of nutrition knowledge, their attention to nutritional labels remained low. Nearly all participants fell within the normal range for stress and anxiety values, with a small proportion of individuals reporting moderate anxiety. In summary, the *t*-test results revealed no significant differences between the control and labeled groups in terms of socio-demographics, food consumption habits, nutrition, and health status.

Data from the food shopping platform included which group a participant was assigned to, the total number of food products chosen by a participant, the total expenditure, and the quantities of calories, fat, carbohydrates, and sodium. The quantity of nutrition elements was obtained from the back-of-pack nutritional content table of each product. The participants, on average, chose six-to-seven food items for their shopping baskets. The majority of individuals self-reported a certainty level of nearly 80% regarding their experimental choices. There are no significant differences in the total expenditure and the count of selected food products between the two groups. However, relative to the control group, individuals in the labeled group exhibited significantly lower levels of calories and sodium in their chosen food items, potentially due to the presence of the traffic light labels.

In addition, this subsection presents the baseline effects of the traffic light labels on the healthiness of food choices, followed by an analysis of their impacts on economic costs. All models are estimated using Equation (1).

The average impacts of the traffic light labels on each of the four nutritional elements—calories, fat, carbohydrates, and sodium—are presented in Table 4. In general, participants in the labeled group, as compared to the control group, exhibited a tendency to select a basket of food items with lower calories, fats, carbohydrates, and sodium content. This observation holds true even when accounting for control variables. Thus, we focus on the model specification with control variables in discussing empirical results, specifically columns (2), (4), (6), and (8) of Table 4.

The results reveal that, on average, the traffic light labels significantly decreased the total calorie intake of an individual in the labeled group by 21%, as compared to the control group. Among the remaining three nutrients, traffic light labels demonstrated the highest effectiveness in reducing sodium intake (30%), followed by fat (25%) and carbohydrates (18%), with statistical significance at the 1% level.

Several studies have suggested that improvements in nutrition induced by labeling often coincide with higher expenditures, disproportionately affecting those with lower incomes in their pursuit of healthier food options [24]. Building upon this, we further investigate the impact of the traffic light labels on the total expenditure (Table 5). Table 5 reveals that incorporating the traffic light labels into prepackaged foods does not lead to an increase in consumers’ food expenditures. Consistent with Muller et al. [24], our study implies that the implementation of MTL does not impose a significant financial burden on consumers, even as it enhances the nutritional quality of their food choices.

### 5.3. Heterogeneity

The utilization of nutrition labels is associated with consumer characteristics. Next, we will investigate how the effects of traffic light labels vary among different genders and levels of nutrition knowledge.

Table 6, Table 7, Table 8 and Table 9 reveal that the effects of MTL vary among groups with different genders and levels of nutritional knowledge. Overall, traffic light labeling has a more significant impact on the female group compared to the male group. In the male subsample, MTL significantly reduced the total calories of food baskets by approximately 39% and total carbohydrate intake by 44%. However, in the female subsample, MTL influenced the intake of all nutritional elements, namely calories, fat, sodium, and carbohydrates. MTL exerted the largest impact on reducing sodium levels in females’ food choices (47.8%), followed by fat (30.4%), carbohydrates (24.9%), and calories (20.5%). This difference in the MTL’s effects between males and females would be associated with the health awareness and nutritional concepts prevalent in different gender groups. Women are more inclined to read nutrition labels compared to men [52,53,54]. Expectedly, MTL has more evident impacts among individuals with lower levels of nutrition knowledge. This observation is grounded in the idea that individuals with higher nutrition knowledge may already possess the necessary information to make informed and healthy food choices, rendering the traffic light label less important for their decision-making process. Table 10 indicates that the MTL scheme has little impact on food expenditures regardless of gender and nutrition knowledge groups.

## 6. Discussions and Conclusions

More than 50 countries worldwide have implemented front-of-pack nutrition labeling, characterized as a collaborative effort involving governments, food industries, and retailers that ultimately guide consumers to healthier food choices [55]. Since half of the adult population in China is overweight or obese, the nutrition labeling scheme, in particular the traffic light nutrition labeling, has growingly received attention and consideration from the Chinese government.

This study offers a novel contribution by assessing the effectiveness of Multiple Traffic Lights (MTL) in guiding Chinese consumers toward healthier food choices through a randomized controlled trial. The insights gained from this research carry implications for the design and implementation of a traffic light labeling scheme in China. Due to the rapid increase in diet-related diseases in recent years, these findings hold significance for addressing and mitigating health concerns in the Chinese population.

The findings reveal that the incorporation of MTL significantly contributes to reducing Chinese consumers’ intake of calories, fat, carbohydrates, and sodium. Specifically, the introduction of traffic light labels resulted in a significant 21% decrease in the total calorie intake for individuals in the labeled group, compared to the control group. Among the other three nutrients, traffic light labels exhibited the greatest effectiveness in reducing sodium intake by 30%, followed by fat at 25% and carbohydrates at 18%. Significantly, our research reveals that traffic light labels improve the healthiness of individuals’ food choices without adding extra costs. Moreover, the impact of MTL differed across groups, showing larger effects on the female subsample and those with lower levels of nutrition knowledge. This underscores the potential of MTL to enhance and complement China’s current back-of-pack nutrition labeling, providing consumers with a more helpful tool for selecting healthy food products. The MTL scheme is especially advantageous for consumers, particularly those who are less knowledgeable about nutrition and nutrition labels since it enables them to choose healthy food products without paying additional costs.

This finding is consistent with several studies showing that front-of-pack labels, such as the MTL scheme, receive more attention from consumers than the existing and classic back-of-pack nutrition panel [56,57,58]. Consumers prefer nutrition labels with graphics and symbols, especially those using colors [52,53]. Front-of-Pack (FOP) schemes, particularly Multiple Traffic Lights (MTL), are found to be visually appealing, friendly, and easy to understand [59,60,61]. Color-coded labels, such as MTL, provide clear visual signals for consumers to recognize healthy and nutritious foods, even without extensive nutrition knowledge. Future research can focus on the application effect of MTL labeling in larger sample sizes and can explore different formats of the MTL scheme, including size, position, and color. Lastly, we acknowledge that this study has some limitations that warrant future research. For example, our focus is on a sample of college students; future research could be extended to investigate the effect of MTL labeling on other demographic groups. Additionally, while we demonstrate the effectiveness of MTL labeling in promoting healthier food choices, other labeling schemes exist. Future research should compare the effectiveness of different nutritional labeling programs to provide a more comprehensive understanding.

## Figures and Tables

**Table 1:** The Standard of Traffic Nutrition Labels.

	Green	Yellow	Red
*Solid food, per 100 g*			
Fat	≤3.0 g	3.0 g–17.5 g	>17.5 g
Carbohydrate	Energy ratio < 40%	Energy ratio 40–70%	Energy ratio > 70%
Sodium	≤120 mg	120 mg–600 mg	≥600 mg
*Liquid food, per 100 mL*			
Fat	≤1.5 g	1.5 g–8.8 g	>8.8 g
Carbohydrate	≤5.0 g	5.0 g–11.3 g	>11.3 g
Sodium	≤120 mg	120 mg–300 mg	≥300 mg

**Table 2:** Variable definition.

Name	Definition
Traffic	=1 if in the nutrition traffic label labeled group, =0 otherwise
Gender	=1 if male, =0 otherwise
Age	Year
Residence	=1 if living in urban, =2 suburb, =3 county, =4 rural
Grade	=1 if fresh year, =2 sophomore, =3 junior, =4 senior, =5 master, =6 doctoral
Height	Meter
Weight	Kilogram
Overweight	=1 if overweight, =0 otherwise
HHsize	Household size
HHinc	Pre-tax total household income in 2021, RMB
Loweight	=1 if on diet, =2 otherwise
Gaweight	=1 if gaining weight, =2 otherwise
Fallergy	=1 if having food allergy, =2 otherwise
Chronic	=1 if having chronic diseases, =2 otherwise
Foodexp	=1 if monthly food expenditure below 600 RMB, =2: 600–1000 RMB, =3: 1000–1500 RMB, =4: 1500–2000 RMB, =5: above 2000 RMB
Outfreq	Monthly online food delivery frequency, 0–100%
Delivfreq	Monthly dining out frequency, 0–100%
Nutrscroe	Nutrition knowledge score, 1–9
Nutriatten	Attention to food nutrition facts panel, 0–100%
Nutricomm	=1 if the current nutrition facts panel needs improvement, =2 otherwise
Pressue	Pressure level, 0–14
Anxiety	Anxiety level, 0–10
Certain	Certainty of food choice in the experiment, 0–100%
Foodnum	Number of food products chosen in the experiment
Texp	Total expenditure in the experiment, RMB
Tcalorie	Total calories from the food products chosen in the experiment, kJ
Tfat	Total fat from the food products chosen in the experiment, gram
Tcarb	Total carbohydrates from the food products chosen in the experiment, gram
Tsodi	Total sodium from the food products chosen in the experiment, milligram

**Table 3:** Balance Test Table for Students in Control and Labeled Groups.

	Traffic = 0 (N = 47)		Traffic = 1 (N = 53)		
Variable	Mean	S.D.	Mean	S.D.	*t*-Test
*Socio-demographics*					
Gender	0.364	0.475	0.375	0.434	0.818
Age	21.380	1.963	21.490	2.259	−0.244
Residen	2.297	1.222	2.075	1.207	0.854
Grade	3.081	1.622	3.075	1.530	0.017
Height	1.671	0.078	1.658	0.066	0.804
Weight	59.660	8.787	56.250	9.705	1.703
Overweight	0.081	0.277	0.094	0.295	−0.215
HHsize	3.892	1.022	3.566	0.844	1.652
HHinc	4.324	1.564	3.925	1.426	1.258
*Usual Food Consumption*					
Foodexp	3.243	0.830	3.130	0.849	1.092
Outfreq	25.700	19.960	27.110	26.650	−0.273
Delivfreq	27.270	26.040	26.640	28.470	0.107
Loweight	1.459	0.505	1.585	0.497	−1.170
Gaweight	1.919	0.277	1.868	0.342	0.751
Fallergy	1.838	0.374	1.943	0.233	−1.649
*Health and Nutrition Status*					
Nutriscore	5.486	1.742	5.245	1.764	0.642
Nutriatten	32.410	28.880	34.910	25.510	−0.433
Nutricomm	1.595	0.498	1.491	0.505	0.968
Chronic	1.946	0.229	1.925	0.267	0.397
Pressure	4.541	3.245	4.755	3.356	−0.302
Anxiety	2.946	2.147	3.340	2.441	−0.790
*Food Choice in the Experiment*					
Certain	79.72	19.79	78.72	19.31	0.235
Foodnum	7.514	3.934	6.755	3.486	0.964
Texp	75.870	63.480	61.340	43.300	1.292
Tcalorie	12,023	8937	9160	5594	1.868 *
Tfat	84.350	64.650	60.780	40.160	2.132
Tcarb	458.700	360.900	361.600	238.200	1.540
Tsodi	3209	2973	2036	1600	2.418 **

Notes: *t*-test scores are obtained from testing equality in each of the variables between the two groups. *, ** denote statistical significance at 10% and 5% level, respectively.

**Table 4:** Nutritional Impacts of Traffic Nutrition Labels.

	Ln(Tcalorie)		Ln(Tfat)		Ln(Tcarb)		Ln(Tsodi)	
	(1)	(2)	(3)	(4)	(5)	(6)	(7)	(8)
Constant	9.190 ***	7.489 ***	4.170 ***	2.344 ***	5.892 ***	4.274 ***	7.716 ***	4.164 ***
	(0.104)	(0.664)	(0.126)	(0.769)	(0.113)	(0.742)	(0.147)	(0.967)
Traffic	−0.286 **	−0.211 **	−0.330 *	−0.258 *	−0.257	−0.183 *	−0.504 **	−0.308 *
	(0.144)	(0.089)	(0.169)	(0.142)	(0.158)	(0.096)	(0.211)	(0.173)
Control		Yes		Yes		Yes		Yes
R^2^	0.041	0.613	0.041	0.483	0.028	0.587	0.057	0.487
Observations	100	100	100	100	100	100	100	100

Notes: The dependent variable takes the natural logarithm form. Control variables are all the variables in Table 2, except for those belonging to food choice in the experiment. *, **, and *** denote statistical significance at 10%, 5%, and 1% level, respectively. Standard errors are in parentheses.

**Table 5:** Economic Impacts of Traffic Nutrition Labels.

	Ln(Texp)	Ln(Texp)
Constant	4.079 ***	2.100 ***
	(0.112)	(0.615)
Traffic	−0.250	−0.121
	(0.162)	(0.090)
Control		Yes
R^2^	0.024	0.687
Observations	100	100

Notes: The dependent variable takes the natural logarithm form. Control variables are all the variables in Table 2, except for those belonging to food choice in the experiment. *, **, and *** denote statistical significance at 10%, 5%, and 1% level, respectively. Standard errors are in parentheses.

**Table 6:** Heterogenous Impacts of Traffic Nutrition Labels on Total Calories.

	Gender		Nutrition Knowledge	
	Female	Male	Below Median	Above Median
Constant	8.352 ***	5.376 ***	7.490 ***	4.423 **
	(0.795)	(1.127)	(0.854)	(1.624)
Traffic	−0.205 *	−0.394 **	−0.185 *	0.106
	(0.103)	(0.179)	(0.098)	(0.588)
Control	Yes	Yes	Yes	Yes
R^2^	0.638	0.793	0.660	0.799
Observations	65	25	71	29

Notes: The dependent variable takes the natural logarithm of Tcalorie. Control variables are all the variables in Table 2, except for those belonging to food choice in the experiment. *, **, and *** denote statistical significance at 10%, 5%, and 1% level, respectively. Standard errors are in parentheses. Below median refers to the subsample whose nutrition knowledge, indicated by Nutriscore, is below the median of the full sample.

**Table 7:** Heterogenous Impacts of Traffic Nutrition Labels on Total Fat.

	Gender		Nutrition Knowledge	
	Female	Male	Below Median	Above Median
Constant	2.696 **	1.850	1.998 *	1.635 *
	(1.073)	(1.436)	(1.074)	(0.853)
Traffic	−0.304 **	−0.384	−0.190 *	−0.374
	(0.134)	(0.356)	(0.098)	(0.517)
Control	Yes	Yes	Yes	Yes
R^2^	0.489	0.577	0.509	0.809
Observations	73	27	71	29

Notes: The dependent variable takes the natural logarithm of Tfat. Control variables are all the variables in Table 2, except for those belonging to food choice in the experiment. *, **, and *** denote statistical significance at 10%, 5%, and 1% level, respectively. Standard errors are in parentheses. Below median refers to the subsample whose nutrition knowledge, indicated by Nutriscore, is below the median of the full sample.

**Table 8:** Heterogenous Impacts of Traffic Nutrition Labels on Total Carbohydrates.

	Gender		Nutrition Knowledge	
	Female	Male	Below Median	Above Median
Constant	5.514 ***	1.322	4.340 ***	0.818 *
	(0.878)	(1.179)	(0.931)	(0.510)
Traffic	−0.249 **	−0.441 **	−0.181 **	0.311
	(0.122)	(0.182)	(0.107)	(0.637)
Control	Yes	Yes	Yes	Yes
R^2^	0.603	0.856	0.634	0.785
Observations	73	27	71	29

Notes: The dependent variable takes the natural logarithm of Tcarb. Control variables are all the variables in Table 2, except for those belonging to food choice in the experiment. *, **, and *** denote statistical significance at 10%, 5%, and 1% level, respectively. Standard errors are in parentheses. Below median refers to the subsample whose nutrition knowledge, indicated by Nutriscore, is below the median of the full sample.

**Table 9:** Heterogenous Impacts of Traffic Nutrition Labels on Total Sodium.

	Gender		Nutrition Knowledge	
	Female	Male	Below Median	Above Median
Constant	4.490 ***	2.712 *	4.043 ***	2.944 **
	(1.422)	(1.406)	(1.188)	(1.296)
Traffic	−0.478 **	−0.099	−0.082	−0.874
	(0.200)	(0.414)	(0.176)	(0.862)
Control	Yes	Yes	Yes	Yes
R^2^	0.481	0.640	0.514	0.802
Observations	73	27	71	29

Notes: The dependent variable takes the natural logarithm of Tsodi. Control variables are all the variables in Table 2, except for those belonging to food choice in the experiment. *, **, and *** denote statistical significance at 10%, 5%, and 1% level, respectively. Standard errors are in parentheses. Below median refers to the subsample whose nutrition knowledge, indicated by Nutriscore, is below the median of the full sample.

**Table 10:** Heterogenous Impacts of Traffic Nutrition Labels on Total Expenditure.

	Gender		Nutrition Knowledge	
	Female	Male	Below Median	Above Median
Constant	2.853 ***	0.431	1.919 ***	−0.194 **
	(0.801)	(1.498)	(0.704)	(0.701)
Traffic	−0.090	−0.254	−0.132	0.207
	(0.105)	(0.315)	(0.097)	(0.714)
Control	Yes	Yes	Yes	Yes
R^2^	0.684	0.782	0.722	0.764
Observations	73	27	71	29

Notes: The dependent variable takes the natural logarithm of Texp. Control variables are all the variables in Table 2, except for those belonging to food choice in the experiment. *, **, and *** denote statistical significance at 10%, 5%, and 1% level, respectively. Standard errors are in parentheses. Below median refers to the subsample whose nutrition knowledge, indicated by Nutriscore, is below the median of the full sample.

## Data Availability

Data will be available upon request.
